# The Awareness and Attitude of Contracted Service Among General Medical Practitioners in Community Health Service Centers in Urban China: A Cross-Sectional Study

**DOI:** 10.3389/fpubh.2021.572311

**Published:** 2021-06-08

**Authors:** Tao Yin, Delu Yin, Huijing He, Xiaoguo Zheng, Ruili Li, Huimin Yang, Lihong Wang, Bowen Chen

**Affiliations:** ^1^Department of Child Health Care, Capital Institute of Pediatrics, Beijing, China; ^2^Department of Epidemiology and Statistics, Institute of Basic Medical Sciences, Chinese Academy of Medical Sciences and Peking Union Medical College, Beijing, China

**Keywords:** contract service, primary care, family doctor, community health, China

## Abstract

This study aims to explore the attitude, willingness, and satisfaction with contracted service (CS) among staff in community health service (CHS) centers in urban China and to explore the associated factors of satisfaction with CS. From August 2016 to July 2017, five CHS centers in three provinces of China were selected. Setting-level information was collected by official document review; and personal information on demographic characteristics, awareness, willingness, and attitude of CS among staff was collected by questionnaire survey. Univariate and multivariable logistic regression models were fitted to explore the associated factors of satisfaction with CS. Multiple correspondence analysis (MCA) was used to visually demonstrate the correlations among category data related with satisfaction with CS. The CS signing rates were 30.78, 12.72, 22.20, 14.32, and 21.19% in the five CHS centers. A total of 286 staff included family doctors (40.91%), nurses (31.12%), and others (27.97%) completed the survey. For the sense of self-worth, 86.01% (246/286) participants hold a positive attitude. The predominant barrier of CS signing was caused by the work pressure due to CS performance assessment (48.60%, 139/286). About 30% of family doctors and nurses reported a heavy work pressure, and more than 30% of doctors had great feeling of fatigue. Notably, 51.69% family doctors would like to change their job in the future. Compared with other staff, family doctors were more likely to be unsatisfied with CS (OR: 2.793, 95% CI: 1.155–6.754, *p* = 0.022). Participants in Sichuan province have lower satisfaction than other places. The MCA yielded similar factors consistent with multivariable results of clustering with different levels of CS satisfaction. Our study revealed that the CS coverage and satisfaction among staff from the primary healthcare system varied geographically and are associated with professional field, workload, and pressure. Measures that aim to promote the stability of primary care human resource should be considered in the future.

## Introduction

The primary healthcare system in China has contributed substantially to disease prevention and control in China for decades (1). In urban areas, family doctors (FDs), also called the family physician or general practitioner, in the community health service (CHS) centers play key role in providing primary healthcare.

In April 2013, the Ministry of Health formally unveiled the contracted service (CS) policy to assist the organization work of primary health services (2). The CS can be defined as contracts endorsed between FDs and urban citizens in the community units, which will facilitate the doctors to provide more tailored medical care; and meanwhile, the citizens can also receive on-time medical service and professional health suggestions. In the mode of CS, residents can choose their FDs voluntarily from local CHS centers. One resident could only sign one health service contract with one FD, but one FD can sign as many contracts with the local residents as is appropriate according to their own wish and capacity (3).

The Chinese government has sped up the development of primary care system from 2009 and announced that by the end of 2020, there will be two to three general medical practitioners for every 10,000 people (4). Despite this encouraging promise, the attitude, willingness, and satisfaction with CS of FDs and other relevant staff in CHS centers are still unclear but are significant in the implementation of CS. There were few studies investigated related to concerns on this topic, most of which used qualitative methods (5) and mainly explored the reasons for certain behavior, or focus on staff in rural areas (5, 6), or from the residents' view to understand their awareness of CS (6–8). These studies provided valuable information on understanding facilitators and barriers of CS implementation in China, but evidence on how the general medical practitioners consider and value CS and what may influence their work enthusiasm and efficacy is sparse.

As reported by a national survey, the primary healthcare doctors in China are experiencing high rate of burnout, with low wages and minimal benefits (1). To better understand the workload, attitude, willingness, and satisfaction with the current CS work experience, providing suggestions to policymakers to better implementing CS, from August 2016 to July 2017, we conducted a cross-sectional survey toward primary healthcare system staff from three provinces in China.

## Methods

### Data Resource and Study Population

Five CHS centers in three provinces were selected as having different economic levels, geographic situations, varied primary healthcare system infrastructure foundations, and investigation feasibility: Xi-hang-gang CHS center and Shuang-zi-qiao CHS center in Sichuan province; Shi-tang CHS center and Ji-mei CHS center in Fujian province; and Yi-cheng CHS center in Shandong province. Selection of areas with varied characteristics may better understand the whole picture of the attitude and workload of staff working on CS. We used a multistage stratified convenience sampling method to select our study participants.

In the first sampling stage, we selected three provinces from 31 provinces, municipalities, and autonomous regions in mainland China based on the feasibility and variations of CHS centers. In the second sampling stage, we selected one or two CHS centers from each province, under the consideration that the chosen CHS centers should cover a large population and has been implemented CS for a relatively long time. In the last stage of sampling, staff working in the selected CHS centers were all invited to participate and underwent a self-reported questionnaire interview independently. The inclusion criteria of the participants were as follows: (1) currently full-time worked in the selected CHS center and (2) being involved in the CS work. The exclusion criteria was that the staff work less than half one year.

The minimum needed sample size was calculated based on an estimated satisfaction proportion of 60%(p). To reach a significance level (alpha) of 0.05 and error tolerance 0.15 p, the estimated minimum sample size was 114. We added an additional 20% to the minimum sample size factoring in possible noncompliance and targeted 143 subjects. Finally, 286 staff from five CHS centers participated in the interview.

Ethical approval was obtained from the Ethical Review Board of Capital Institute of Pediatrics. All participants provided written informed consent before the survey.

### Measurement

Setting-level information was derived from official documents in each CHS center or from the official website of local health committee. The contents include the population coverage in each study setting, the coverage of chronic disease (such as hypertension and diabetes) patients signing CS, and the number of medical staff with different professional fields.

Individual-level data were collected through structured questionnaire interview from August 2016 to July 2017. The questionnaire was self-reported anonymously by participants to avoid potential influence by the investigator. The questionnaire includes three parts. The first part is about the demographic characteristics and basic information about the participants, such as age, educational level [divided into three groups, high school, junior college (which is a 2-year professional training program similar with college study), and college], professional field, and work experience. The second part is about work pressure and feeling of fatigue due to the implementation of CS. The third part is about the staff's attitude and satisfaction with CS. Several relevant questions were designed to reflect the satisfaction with CS among the study participants, including aspects of their satisfaction with the environment, the financial support, the sense of self-worth, the patient–physician relationship, and their long-term willingness of being involved in the CS (descripted by the question “Would you like to change your job in the future? If yes, how strong your willingness is?”). Moreover, we added one interesting question of “Would you like your child to be a family doctor when they grow up?” to understand how they value the primary healthcare. The satisfaction or willingness with CS and its relevant aspects was measured with a five-point Likert scale (1 = very unsatisfied/never to 5 = very satisfied/very likely).

### Statistical Analyses

Continuous variables were presented as mean and standard deviation (SD) and categorical data as number and percentage. A *p*-value < 0.05 (two-tailed) was considered statistically significant. Radar chart was used to demonstrate the barriers in implementing CS and the factors that contribute mostly to CS signing. Chi-square tests for categorized data were used to compare characteristics of participants.

Univariate and multivariable logistic regression models were used to examine the potential influencing factors of satisfaction with CS among the study population. In this process, we fitted an unadjusted model and then gradually adjusted for demographic characteristics (age and gender), work experience, work pressure, and the feeling of fatigue to estimate the association between satisfaction and those factors. The dependent variable in the logistic regression model was whether the staff was satisfied with CS. To fit the dichotomous logistic model, we integrated “very satisfied” and “satisfied” as one category and “not satisfied” and “neutral or not clear” as another category. Similarly, in the further analysis of study participants' preference of their offspring's career choice, the answers of “Yes, I will definitely support my child if he/she would like to be a family doctor” and “Yes, I may support my child if he/she would like to be a family doctor” were combined into one category, and the answers of negative attitude such as “I don't want my child to be a family doctor” and “I will be strongly against if my child would like to be a family doctor” were combined into one category. Multiple correspondence analysis (MCA) was used to study cross-frequency tables (contingency tables) that explore the simultaneous relationships between variables (9). The results were presented on the basis of the relative positions of the points of each category distributed in the two-dimensional coordinate system; as categories become more similar in distribution, the closer (distance between the points) they are presented in space (9, 10).

All statistical analyses were performed using SAS 9.4 (SAS Institute Inc. Cary, NC, USA).

## Results

### Basic Characteristics of the Selected Community Health Service Centers and Study Participants

The basic characteristics of the five selected CHS centers from Shandong, Sichuan, and Fujian provinces in China are shown in Table 1. The population coverage by each CHS center is around 100,000, but the proportion of population signed CS was low, with the highest CS signing proportion of 30.78% in Xi-hang-gang CHS center in Sichuan province and the lowest proportion of 12.72% in Shi-tang CHS in Fujian province. But the coverage of CS for common chronic disease, such as diabetes and hypertension, was higher at around 50% in Sichuan and Fujian provinces but lower in Shandong province (around 10%). The number of FDs provided CS for every 100,000 residents were much higher in Yi-cheng center in Shandong province, which is 88, compared with the other four centers of 28, 23, 17, and 24, respectively (Table 1).

**Table 1:** Basic characteristics of the five selected CHS centers from three provinces in China, 2016.

**CHS centers**	**1**	**2**	**3**	**4**	**5**
Population coverage	141,721	100,800	28,752	143,850	86,221
Population covered by CS (*n*, %)	43,627 (30.78)	12,817 (12.72)	6,382 (22.20)	20,606 (14.32)	18,267 (21.19)
DM patients	10,495	2,873	1,230	6,543	7,063
DM patients covered by CS (*n*, %)	5,812 (55.38)	1,426 (49.63)	877 (71.30)	4,105 (62.74)	740 (10.48)
HTN patients	20,342	7,596	3,459	11,564	18,324
HTN patients covered by CS (*n*, %)	12,078 (59.37)	3,172 (41.76)	2,116 (61.17)	8,256 (71.39)	2,203 (12.02)
Population diagnosed as both DM and HTN	5,043	1,488	759	2,687	3,960
DM and HTN patients covered by CS (*n*, %)	2,761 (54.75)	856 (57.53)	512 (67.46)	1,733 (64.50)	385 (9.72)
Number of staff (% of participants)	140 (53.6)	80 (91.3)	33 (60.6)	69 (33.3)	202 (46.5)
Number of medical staff (% of participants)	124 (52.4)	59 (81.4)	28 (57.1)	59 (35.6)	170 (45.3)
Number of doctors (% of participants)	47 (63.8)	36 (97.2)	12 (83.3)	17 (23.5)	76 (50.0)
Number of nurses (% of participants)	49 (57.1)	13 (100)	4 (100)	30 (46.7)	74 (40.5)
Number of public health staff (% of participants)	24 (54.2)	13 (100)	5 (80)	14 (100)	14 (46.7)
Number of family doctors provided CS (% of participants)	40 (75.0)	23 (100)	5 (100)	35 (80)	76 (78.9)
Number of family doctors who provide CS for every 100,000 residents	28	23	17	24	88

*Medical staff included doctors, nurses, public health staff and pharmacies*.

*CHS, community health service; CS, contracted service; HTN, hypertension; DM, diabetes; FD, family doctor*.

*(1) Xi-hang-gang CHS center in Sichuan province; (2) Shi-tang CHS center in Fujian province; (3) Ji-mei CHS center in Fujian province; (4) Shuang-zi-qiao CHS center in Sichuan province; (5) Yi-cheng CHS center in Shandong province*.

The basic characteristics of study participants stratified by sex are shown in Table 2. A total of 286 staff completed the questionnaire survey, of which 197 (68.88%) were female. Most of the participants were younger than 40 (75.53%), had at least junior college educational attainment (94.06%), and had more than 5 years of work experience (65.73%); 40.91% were FDs; and 31.12% were nurses (Table 2).

**Table 2:** Sociodemographic and professional characteristics of study participants from five community health service centers in China, 2016.

**Variables**	**Male (*****n*** **=** **89)**		**Female (*****n*** **=** **197)**		***P***	**Overall (*****n*** **=** **286)**	
Age (year, mean, SD)	36.18	7.25	33.29	7.22	0.002	34.18	7.34
**Age (year**, ***n*****, %)**							
19–29	15	16.85	74	37.56	0.002	89	31.12
30–39	45	50.56	82	41.62		127	44.41
40–49	22	24.72	34	17.26		56	19.58
50–58	7	7.87	7	3.55		14	4.90
**Areas (*****n*****, %)**							
Fujian	31	34.83	62	31.47	<0.001	93	32.52
Shandong	42	47.19	52	26.40		94	32.87
Sichuan	16	17.98	83	42.13		99	34.62
CHS centers^*^ **(*****n*****, %)**							
1	15	16.85	61	30.96	<0.001	76	26.57
2	23	25.84	50	25.38		73	25.52
3	8	8.99	12	6.09		20	6.99
4	1	1.12	22	11.17		23	8.04
5	42	47.19	52	26.40		94	32.87
**Education (*****n*****, %)**							
High school	4	4.49	12	6.09	0.510	16	5.59
Junior College	41	46.07	101	51.27		142	49.65
College or above	44	49.44	83	42.13		127	44.41
**Work experience as CHS staff [years, (*****n*****, %)]**							
0.5–1.9	7	7.87	25	12.69	0.043	32	11.19
2–4.9	17	19.10	49	24.87		66	23.08
5–9.9	20	22.47	58	29.44		78	27.27
10–26	45	50.56	65	32.99		110	38.46
**Professional field (*****n*****, %)**							
Clinical service	57	64.04	60	30.46	<0.001	117	40.91
Public health	4	4.49	8	4.06		12	4.20
Nursing or medical care	4	4.49	85	43.15		89	31.12
Pharmacy	5	5.62	14	7.11		19	6.64
Others	19	21.35	29	14.72		48	16.78

*CHS, community health service*.

* *(1) Xi-hang-gang CHS center in Sichuan province; (2) Shi-tang CHS center in Fujian province; (3) Ji-mei CHS center in Fujian province; (4) Yi-cheng CHS center in Shandong province; (5) Shuang-zi-qiao CHS center in Sichuan province*.

### The Awareness and Attitude Toward Contracted Service

**Figure 1:**
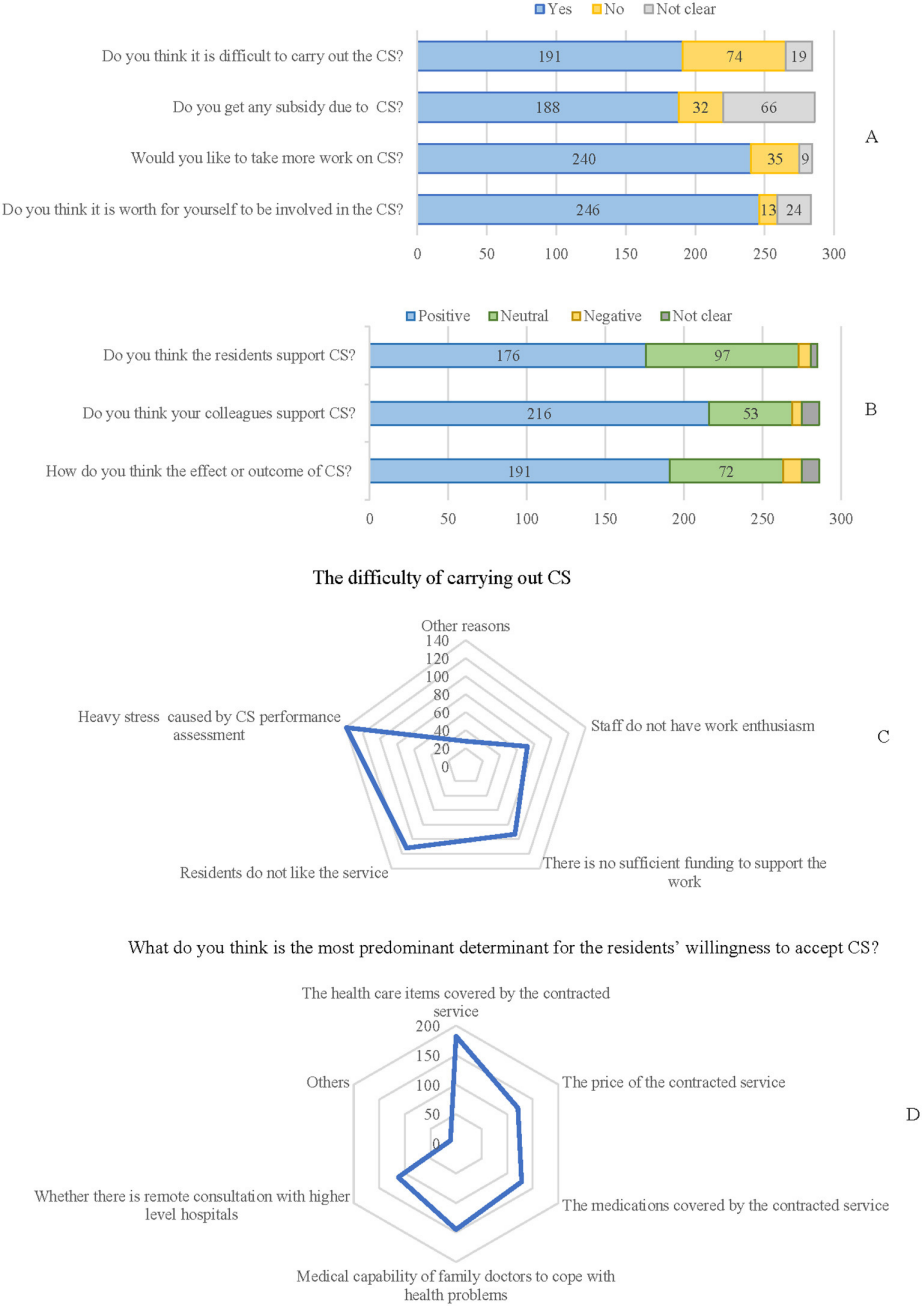
The awareness, perspectives, and attitude of contracted service among the general medical practitioners. **(A,B)** The awareness, attitude, and perspectives toward contracted service (CS) among participants. **(C)** The difficulty of carrying out CS. **(D)** Predominant determinant factors influencing the willingness of CS signing from the perspective of medical staff.

The awareness and attitude toward CS among staff in the selected CHS centers are presented in Figure 1. In Figure 1A, 66.78% (191/286) of the participants agree that there were difficulties in carrying out CS, and 65.73% (188/286) have subsidy because of CS signing. For the sense of self-worth, 86.01% (246/286) of the staff hold a positive attitude. As presented in Figure 1B, most of the staff (61.54%) thought the residents covered by their CHS center support signing CS, and 75.52% thought their colleagues support CS. For the subjective assessment of the effect of CS, 66.78% (191/286) of the staff hold a positive attitude, and only 4.20% (12/286) have a negative attitude.

Since the coverage of CS signing among the common population was not high (around 50% in the selected centers), the difficulty of signing CS was investigated and presented in Figure 1C. The results showed that the predominant factor influencing CS signing was work pressure due to CS performance assessment (48.60%, 139/286), which means that the center should reach a certain proportion of CS signing to meet the strategy established by the National Health Committee. Others were mainly external factors, such as no sufficient financial support (32.52%, 93/286) or residents do not prefer CS (39.16%, 112/286). From the perspective of health providers, the most predominant factor influencing resident's willingness to sign CS was the healthcare items covered by the CS (63.64%, 182/286) (Figure 1D).

### The Workload and Satisfaction With Community Health Service

The workload and satisfaction with current CHS among the study participants who have taken charge of CS, stratified by professional fields, are shown in Table 3. About 30% of doctors and nurses reported a heavy work pressure, and more than 30% of doctors had great feeling of fatigue.

**Table 3:** Workload and satisfaction with current CHS among the general medical practitioners who had been involved in the CS.

	**Doctors**		**Nurses**		**Others**		**p**	**Overall**	
**Additional worktime due to CS**	*n*	%	*n*	%	*n*	%	0.686	n	%
Large >1 h/day	38	42.70	50	42.74	30	37.97		119	41.61
Moderate (<1 h/day)	43	48.31	56	47.86	37	46.84		136	47.55
None	6	6.74	7	5.98	9	11.39		22	7.69
**Work pressure assessment**									
Heavy	30	33.71	41	35.04	13	16.46	<0.001	84	29.37^b^
Moderate	32	35.96	62	52.99	41	51.90		135	47.20
Light	27	30.34	13	11.11	25	31.65		65	22.73
**Feeling of fatigue**									
Heavy	31	34.83	28	23.93	11	13.92	0.002	70	24.48^a^
Moderate	32	35.96	68	58.12	43	54.43		143	50.00
Light	26	29.21	21	17.94	25	31.65		72	24.55
**Satisfaction with working environment**									
Very satisfied	9	10.11	16	13.68	13	16.46	0.134	38	13.29
Satisfied	53	59.55	65	55.56	50	63.29		169	59.09
Neutral	23	25.84	30	25.64	12	15.19		65	22.73
Unsatisfied/very unsatisfied	2	2.25	6	5.12	2	2.53		10	3.50
**Satisfaction with patient–physician relationship**									
Very satisfied	10	11.24	15	12.82	18	22.78	0.015	43	15.03^b^
Satisfied	52	58.43	77	65.81	50	63.29		179	62.59
Neutral	24	26.97	19	16.24	8	10.13		52	18.18
Unsatisfied/very unsatisfied	3	3.37	6	5.12	0	0		9	3.15
**Satisfaction with CS**									
Very satisfied	13	14.61	17	14.53	15	18.99	0.077	45	15.73
Satisfied	45	50.56	65	55.56	52	65.82		162	56.64
Neutral	27	30.34	28	23.93	12	15.19		68	23.78
Unsatisfied/very unsatisfied	3	3.37	7	5.98	0	0		10	3.50
**Willingness to change job**									
Yes	46	51.69	46	39.32	32	40.51	0.134	125	43.71
No	42	47.19	69	58.97	46	58.23		157	54.90
Would you like your child to be a family doctor?^*^									
Yes	33	37.08	45	38.46	52	65.82	<0.001	130	45.45^a^
No	55	61.80	67	57.26	23	29.11		146	51.05

*CS, contracted service*.

* *For participants who had no child, or their child is under 18 years old*.

a *p < 0.01*.

b *p < 0.05*.

**Figure 2:**
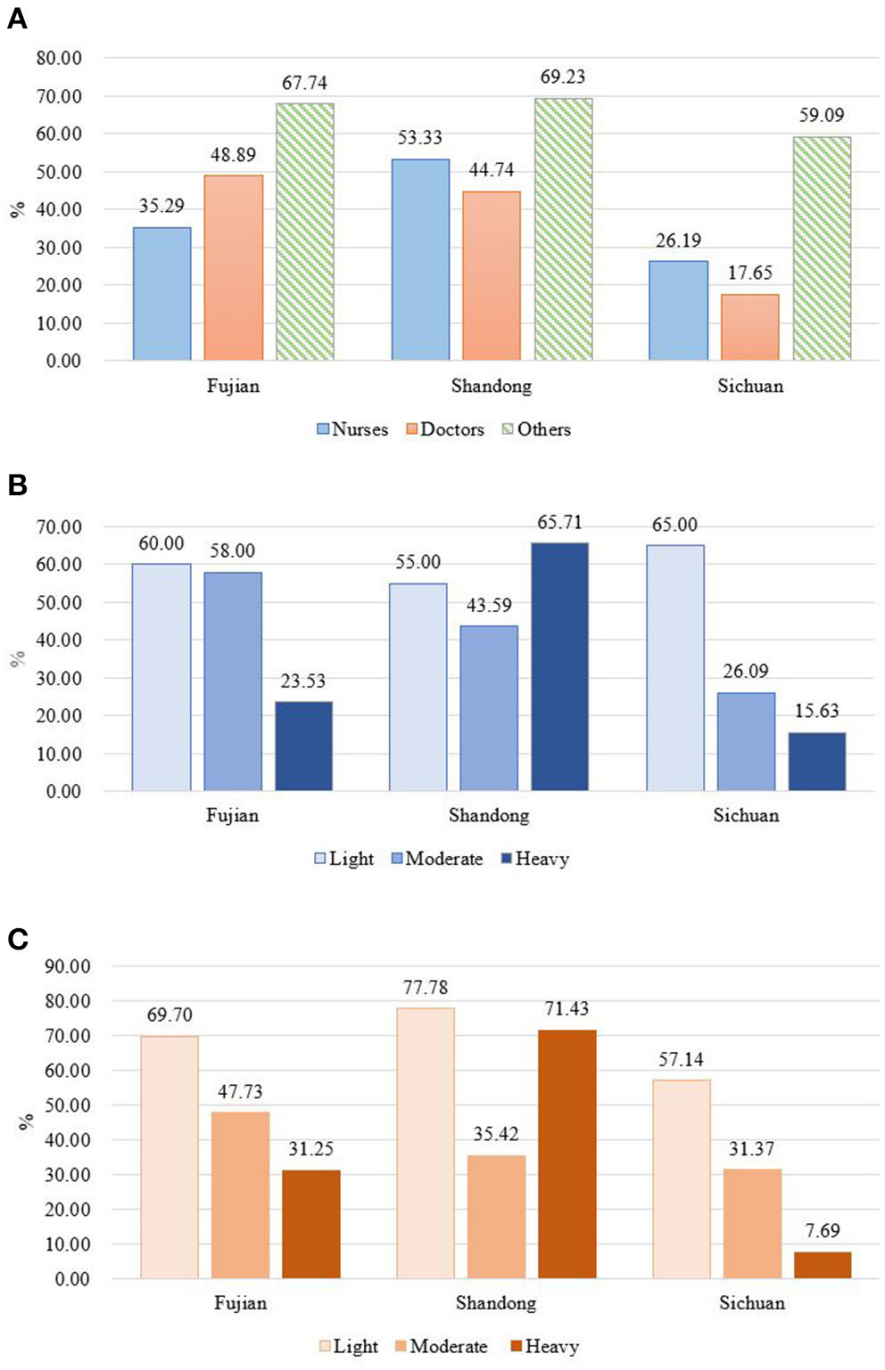
The proportion of staff who would like their child to be a family doctor, stratified by study areas, self-report work pressure, and feeling of fatigue in different study areas. **(A)** Stratified by professional fields; **(B)** stratified by self-report work pressure; **(C)** stratified by self-report feeling of fatigue.

Interestingly, when the participants were asked if they want their child to be an FD, only 45.45% said yes. This proportion in FDs was the lowest, only 37.08%. This willingness or wish was related with professional fields, work pressure, and feeling of fatigue (Figure 2). In all the study sites, doctors and nurses who provided direct medical service to patients had lower positive willingness proportion than the others. In Sichuan, only 17.65% of nurses and 26.19% of doctors wanted their child to be an FD. Work pressure and feeling of fatigue appeared negatively associated with this willingness in Fujian and Sichuan provinces but showed an opposite result in Shandong province, where the work pressure and feeling of fatigue of staff seemed to have no influence on their willingness of offspring career seeking.

### Associated Factors of Satisfaction With Contracted Service

**Figure 3:**
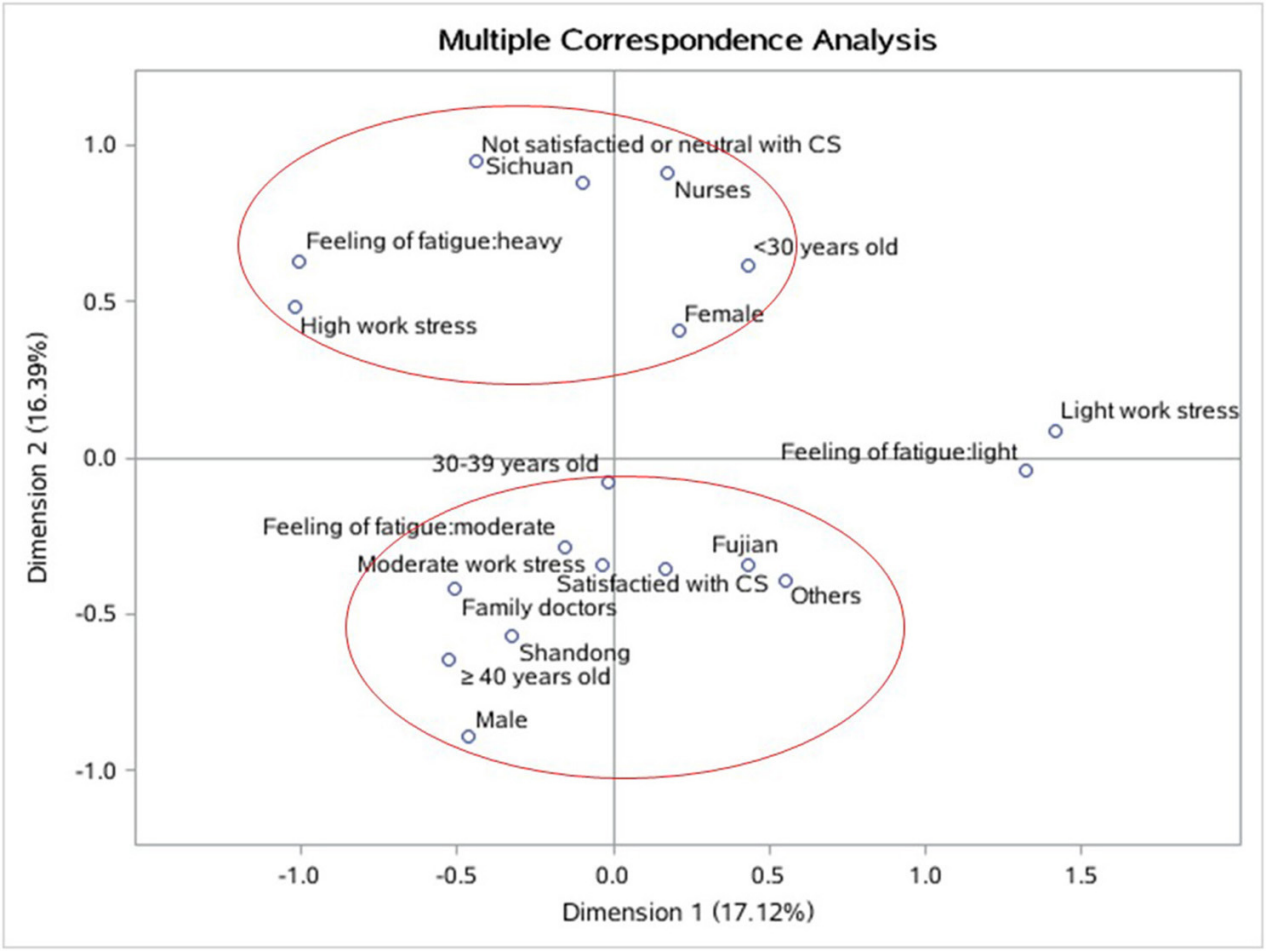
The multiple correspondence analysis (MCA) among satisfaction with CS and its associated factors. Red rings circle the factors that cluster with different levels of satisfaction with CS. The lowest distance was considered as highest degree of similarity in the corresponding dimension. CS, contracted service.

The potential influence factors of satisfaction with CS services among participants are shown in Table 4. Compared with the other staff, doctors were more likely to be unsatisfied with CS (OR: 2.793, 95% CI: 1.155–6.754, *p* = 0.022). Participants in Sichuan province had lower satisfaction than other places, while those from Shandong have higher satisfaction. Other covariates, such as age group, sex, educational level, work pressure, or feeling of fatigue, were not found be associated with the satisfaction of CS. The MCA also illustrated factors that cluster with different levels of satisfaction with CS (Figure 3). The lowest distance was considered as the highest degree of similarity in the corresponding dimension, and the plots demonstrated that not satisfied with CS was strongly associated with study areas (Sichuan province) and professional field (nurse), which was consistent with the logistic regression model; moderate work stress and moderate feeling of fatigue were closely associated with feeling satisfied with CS.

**Table 4:** Potential influence factors of satisfaction with CS among general medical practitioners^*^.

	**Satisfaction with CS**											
	**Yes**		**Neutral or No**		**C-OR**	**95% CI**		***P***	**A-OR**	**95% CI**		***P***
**Professional field**	***n***	**%**	***n***	**%**								
Doctors	82	70.09	35	29.91	2.383	1.148	4.949	0.012	2.793	1.155	6.754	0.023
Nurses	58	65.17	30	33.71	2.888	1.356	6.151	0.006	1.977	0.770	5.075	0.157
Others	67	84.81	12	15.19	1	NA	NA	NA	1	NA	NA	NA
**Age group**												
19–29	58	65.17	30	33.71	1	NA	NA	NA	1	NA	NA	NA
30–39	92	72.44	35	27.56	2.268	1.075	4.784	0.032	1.929	0.692	5.379	0.209
40–58	57	81.43	13	18.57	1.668	0.814	3.417	0.162	1.585	0.606	4.148	0.348
**Sex**												
Male	74	83.15	15	16.85	1	NA	NA	NA	1	NA	NA	NA
Female	133	67.51	63	31.98	2.337	1.244	4.391	0.008	1.663	0.736	3.761	0.222
**Study areas**												
Fujian	75	80.65	18	19.35	1	NA	NA	NA	1	NA	NA	NA
Shandong	88	93.62	6	6.38	0.284	0.107	0.752	0.011	0.187	0.062	0.563	0.003
Sichuan	44	44.44	54	54.55	5.114	2.668	9.800	<0.001	3.668	1.752	7.680	0.001
**Education**												
High school	12	75.00	4	25.00	1	NA	NA	NA	1	NA	NA	NA
Junior college	100	70.42	41	28.87	1.230	0.375	4.037	0.733	0.836	0.194	3.614	0.811
College or above	95	74.80	32	25.20	1.011	0.304	3.356	0.986	0.502	0.112	2.245	0.367
**Work pressure**												
Light	56	86.15	8	12.31	1	NA	NA	NA	1	NA	NA	NA
Moderate	98	72.59	37	27.41	2.643	1.150	6.071	0.022	1.534	0.405	5.808	0.529
Heavy	52	61.90	32	38.10	4.307	1.819	10.197	0.001	3.218	0.648	15.977	0.153
**Feeling of fatigue**												
Light	62	86.11	9	12.50	1	NA	NA	NA	1	NA	NA	NA
Moderate	100	69.93	43	30.07	2.962	1.351	6.495	0.007	2.458	0.667	9.056	0.176
Heavy	45	64.29	25	35.71	3.827	1.631	8.981	0.002	1.924	0.386	9.583	0.424

*C-OR, crude odds ratio without adjustment for covariates; A-OR, adjusted odds ratio, adjusted for covariates listed in the table; CS, contracted service*.

* *The dependent variable was whether satisfied with the current CS, yes = 0, no or neutral = 1*.

## Discussion

In this study, we explored the awareness, willingness, attitude, and satisfaction with CS provided by FDs in five urban areas in China. With heavy workload and feeling of fatigue, the satisfaction with CS in medical service providers, such as FDs and nurses, were relatively low. However, most participants in this study had acknowledged that CS was necessary for better primary care practice in community health centers, which was reflected by high sense of self-worth and the willingness of carrying out CS. Since CS is still in its initial stage in China, there are still concerns on its effectiveness and practices that needed to be further understood. Evidence from the perspective of health service providers is sparse; thus, our study added useful information and may provide some enlightenment for policymakers to initiate more tailored strategies to enhance the CS implementation.

CS for improving primary healthcare is implemented not only in China but also in other places, such as Bangladesh (11), Tanzanian (12), and Brazil (13). However, there are obvious differences in CS between these countries and China. CS in the abovementioned countries are provided by non-governmental organizations (NGOs) who signed the contract with the local government, but not with the citizen. Caring for a fifth of the world's population, which is aging and has a growing prevalence of chronic disease, China has made remarkable progress in strengthening its primary healthcare systems (1). There are mainly two kinds of institutes providing primary health in China: community health centers in urban areas and town-ship hospitals in rural areas. As the process of urbanization is accelerating, the development of CHS centers is now facing a large challenge: uneven regional distribution of doctors, the commonly reported intentions to quit practice, no legally mandated social benefits, etc. (1).

China adopted FDs to help achieve “Health China 2030” by providing comprehensive, life span CS (14). However, there is disparity between actual and targeted situation because residents are used to visiting specialists in tier two or tier three hospitals regardless of their disease severity (14). Our study revealed that the CS signing proportion was only around 20% in the selected five CHS centers. Most of the population covered by the CHS centers did not sign CS with FDs. The proportion of signed CS in this study is much lower than that of the report by Zhao et al. (56.67% in the urban elders in Zhejiang province of China) (3). This may be attributed to the heterogeneity of study population. The center-level data (presented in Table 1) and previous studies (15, 16) suggested that people with chronic disease are more likely to sign CS. Elders who may be more vulnerable to suffering chronic disease could have higher signing prevalence. Geographic variety may also contribute to differences in CS signing. The strategies and subsidy for primary health in different areas are not universal but influenced by local health policies and socioeconomic status (17). People used to seek medical care at large hospitals even for simple health problems possibly because of their low trust of CHS or outdated equipment there (15, 18, 19); therefore, areas with higher large hospital coverage for local population may have lower CS signing proportion. The first study site, Xi-hang-gang CHS center, is close to the airport and is an urban–rural junction area. On the contrary, Shuang-zi-qiao CHS center is in downtown Chengdu city of Sichuan province, with large hospitals nearby. This may partially explain the remarkable difference in the signing proportion between the two CHS centers in the same city (30.78 vs. 14.32%).

Although CS has been emphasized on the national level, barriers in the implementation cannot be ignored. Revealed by our study, more than 60% (191/286) of the staff in the study CHS centers felt difficulty in carrying out CS. The predominant reason was from the work pressure caused by CS performance assessment, and this can also be reflected by the fact that more than one-third of doctors involved in the interview felt fatigue. Work-related stress in primary care physicians has been recognized for decades all over the world, such as in the USA, the UK, and Germany, with different primary care systems (20, 21). In China, the study of Zhou et al. (2) also revealed that primary health providers, which were village doctors in their study, thought that their workload had increased by providing public healthcare after the CS was implemented. The increased workload is also partially attributed to the staff shortage. Contrary to increased work performance assessment, the funding support is not sufficient (22). The consequence of these problems was the instability of human resource in primary care providers, especially for those who provided CS directly. Data in our study showed that more than half of the doctors and more than one-third of the nurses wanted to change their jobs. The quality of CS depends on health providers in sufficient numbers (23). An unstable workforce is not conducive for the sustainable development of primary healthcare (24). The preference of their offspring's job choice also reflected the difficulty or hardship in working in CHS settings.

Interestingly, our data showed that the satisfaction with CS was high (72.37%). When we carried out this survey, the satisfaction mainly referred to the content, rather than the difficulty of implementation or the sense of necessity of CS. Although barriers of signing CS exist, most staff, including FDs and nurses, support CS. The findings of the study suggested that improvement in performance assessment and promotion of financial support for CS may play key roles in the work passion and the human resource stability.

In line with previous studies (7, 8), healthcare contents and service coverage were found to be the most predominant influencing factors for residents to sign CS. Based on the study of Shang et al., the top needs of the residents in CS were health consultation, regular physical examination, and increasing the proportion of medical insurance reimbursements (7). In some areas of China, the proportion of medical insurance reimbursement is related with the level of medical facilities. For example, in Beijing, to encourage the utilization of primary healthcare, the reimbursement proportion of outpatient cost in primary healthcare settings is 90%, much higher than that in high-level hospitals (25). This policy could to some extent expand the coverage of CS.

In the form of contractual services, FDs provide safe, effective, continuous, and basic medical and public health services (26). The study of Chen et al. reported that the willingness-to-pay of general practitioners using CS among elderly empty nesters was high and suggested that the implementation of CS in developing areas is feasible (26). With signed CS, FDs could be collected more tightly with residents, especially those with chronic disease and those needing close monitoring of physical conditions. Being covered by CS could also contribute to health promotion. A study in Shanghai revealed that older people who signed CS had higher proportion of chronic disease self-management behaviors than their counterparts (27).

Contrary to the elders, most young people are able to update their health status by routine physical checkups paid by their employer rather than go to CHS for health examinations. It is important to enhance the awareness of CS embedded in the CHS to young residents to expand the coverage of CS signing and, thus, provide health consultation and promotion to a wider population and initiate health-related lifestyle interventions before chronic disease onset.

The limitations of this study should be acknowledged. There could be sociocultural factors that influence the participants' attitude toward CS; but limited by the quantitative study design, we were not able to learn this in this study. Thus, qualitative study using in-depth interview is needed in our future work. Moreover, the information on factors associated with CS signing should also be collected from the residents to make the understanding more comprehensive.

## Conclusions

This study revealed some findings on CS provided by urban CHS centers in China, from the perspectives of primary health providers. There are geographic and center-varied coverage of CS signing proportion in the study sites. The satisfaction with CS among CHS center staff was high, but most of them had the feeling of fatigue and high or moderate work pressure. Remarkably, under the pressure of CS signing performance assessment and other factors, more than half FDs had the willingness to change their job. Measures and strategies that aim to promote the stability of the human resource of primary healthcare system should be considered in the future.

## Data Availability Statement

The raw data supporting the conclusions of this article will be made available by the authors, without undue reservation.

## Ethics Statement

The studies involving human participants were reviewed and approved by the Ethical Review Board of Capital Institute of Pediatrics. The patients/participants provided their written informed consent to participate in this study.

## Author Contributions

BC and HH: conceptualization. TY, DY, and HH: methodology. HH and DY: software, validation, and writing—review and editing. HH and TY: formal analysis, resources, and funding acquisition. TY, DY, XZ, RL, HY, LW, and BC: investigation. TY: writing—original draft preparation, visualization, and data curation. HH and BC: supervision. BC: project administration. All authors agree to be accountable for the content of the work.

## Conflict of Interest

The authors declare that the research was conducted in the absence of any commercial or financial relationships that could be construed as a potential conflict of interest.
